# Methyl 3′-benzyl-4′-(2,4-di­chloro­phen­yl)-1′-methyl-2-oxo­spiro­[indoline-3,2′-pyrrolidine]-3′-carboxyl­ate

**DOI:** 10.1107/S1600536814003523

**Published:** 2014-02-22

**Authors:** S. Karthikeyan, P. Narayanan, K. Sethusankar, Anthonisamy Devaraj, Manickam Bakthadoss

**Affiliations:** a Department of Physics, RKM Vivekananda College (Autonomous), Chennai 600 004, India; b Department of Organic Chemistry, University of Madras, Maraimalai Campus, Chennai 600 025, India

## Abstract

In the title compound, C_27_H_24_Cl_2_N_2_O_3_, the indole ring system is essentially planar, with a maximum deviation of 0.082 (2) Å for the carbonyl C atom. It makes a dihedral angle of 88.53 (6)° with the mean plane of the 4-methyl­pyrrolidine ring, which adopts an envelope conformation with the N atom at the flap position. The mol­ecular structure is stabilized by intra­molecular C—H⋯O hydrogen bonds, which generate *S*(6) and *S*(7) ring motifs, and an intra­molecular π–π inter­action involving the benzyl and di­chloro-substituted benzene rings [centroid–centroid distance = 3.6291 (11) Å]. In the crystal, mol­ecules are linked *via* N—H⋯O hydrogen bonds, forming *C*(7) chains running parallel to [10-1].

## Related literature   

For the biological activity of spiro-oxindole derivatives, see: Hilton *et al.* (2000 ▶). For a related crystal structure, see: Karthikeyan *et al.* (2014 ▶). For puckering parameters, see: Cremer & Pople (1975 ▶). For graph-set motifs, see: Bernstein *et al.* (1995 ▶). For bond-length distortions in small rings, see: Allen (1981 ▶).
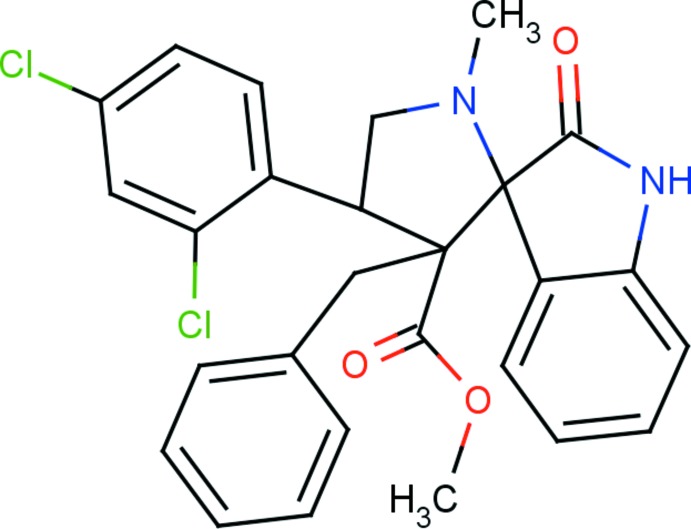


## Experimental   

### 

#### Crystal data  

C_27_H_24_Cl_2_N_2_O_3_
*M* *_r_* = 495.38
Monoclinic, 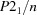

*a* = 12.7051 (5) Å
*b* = 14.1724 (6) Å
*c* = 14.0322 (6) Å
β = 109.424 (2)°
*V* = 2382.85 (17) Å^3^
*Z* = 4
Mo *K*α radiation
μ = 0.31 mm^−1^
*T* = 293 K
0.30 × 0.28 × 0.25 mm

#### Data collection  

Bruker Kappa APEXII CCD diffractometer
27865 measured reflections
6466 independent reflections
4356 reflections with *I* > 2σ(*I*)
*R*_int_ = 0.028

#### Refinement  

*R*[*F* ^2^ > 2σ(*F* ^2^)] = 0.044
*wR*(*F* ^2^) = 0.124
*S* = 1.00
6466 reflections
309 parameters
H-atom parameters constrained
Δρ_max_ = 0.41 e Å^−3^
Δρ_min_ = −0.49 e Å^−3^

### 

Data collection: *APEX2* (Bruker, 2008 ▶); cell refinement: *SAINT* (Bruker, 2008 ▶); data reduction: *SAINT*; program(s) used to solve structure: *SHELXS97* (Sheldrick, 2008 ▶); program(s) used to refine structure: *SHELXL97* (Sheldrick, 2008 ▶); molecular graphics: *ORTEP-3 for Windows* (Farrugia, 2012 ▶); software used to prepare material for publication: *SHELXL97* and *PLATON* (Spek, 2009 ▶).

## Supplementary Material

Crystal structure: contains datablock(s) global, I. DOI: 10.1107/S1600536814003523/su2699sup1.cif

Structure factors: contains datablock(s) I. DOI: 10.1107/S1600536814003523/su2699Isup2.hkl

Supporting information file. DOI: 10.1107/S1600536814003523/su2699Isup3.cml

CCDC reference: 888482

Additional supporting information:  crystallographic information; 3D view; checkCIF report

## Figures and Tables

**Table 1:** Hydrogen-bond geometry (Å, °)

*D*—H⋯*A*	*D*—H	H⋯*A*	*D*⋯*A*	*D*—H⋯*A*
C18—H18*B*⋯O1	0.97	2.31	3.046 (2)	132
C24—H24⋯O3	0.93	2.52	3.155 (2)	126
N2—H2*A*⋯O2^i^	0.86	2.07	2.924 (2)	170

Symmetry code: (i) 
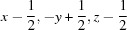
.

**Fig. 1.**
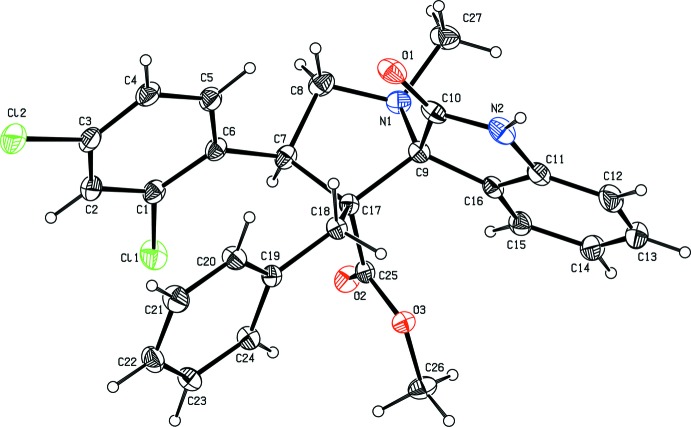
The molecular structure of the title molecule, with atom labelling. Displacement ellipsoids are drawn at 30% probability level.

**Fig. 2.**
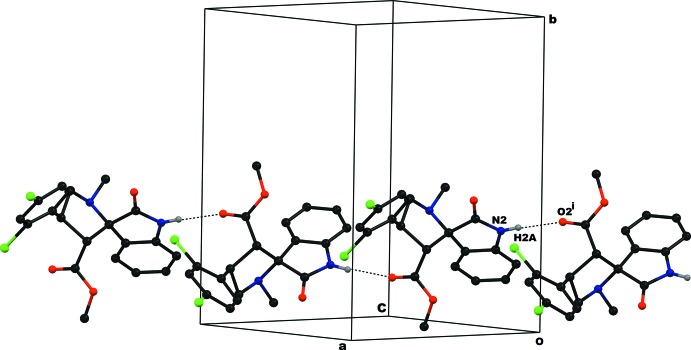
A partial view of the crystal packing of the title compound, showing the formation of infinite C(7) chains. The dashed lines indicate N—H···O hydrogen bonds - see Table 1 for details.

## supplementary crystallographic information

### 1. Comment

The derivatives of spiro-oxindole ring systems are used as antimicrobial, antitumor agents and as inhibitors of the human NKI receptor besides being found in a number of alkaloids like horsifiline, spirotryprostatin and (+)elacomine (Hilton *et al.*, 2000).

The molecular structure of the title compound is illustrated in Fig 1. In the molecule, there are C—H···O hydrogen bonds forming *S*(6) and *S*(7) ring motifs (Bernstein *et al.*, 1995), and a π···π interaction [*Cg*(1)···*Cg*(2) = 3.6291 (11) Å, where *Cg*1 and *Cg*2 are the centroids of rings C1—C6 and C19—C24, respectively]. The indole ring system is essentially planar with a maximum deviation of 0.082 (2) Å for atom C10. The mean plane of this indole ring system forms a dihedral angle of 88.53 (6)° with the 4-methylpyrrolidine ring mean plane. The latter forms a dihedral angle of 83.37 (9)° with the benzyl ring which shows that they are almost orthogonal. Atom O1 significantly deviates from the mean plane of the indole ring system by -0.2251 (15) Å. The molecular dimensions in the title compound are in excellent agreement with those reported for the 3-bromophenyl derivative (Karthikeyan *et al.*, 2014).

The spiro-pyrrolidine ring (N1/C7-C9/C17) adopts an envelope conformation with atom N1 at the flap. The distance to the flap position from the mean plane of the four C atoms is 0.2476 (16) Å; the ring puckering parameters (Cremer & Pople, 1975) are Q_2_ = 0.3917 (18) Å and φ_2_ = 2.8 (3)°. The central spiro-pyrrolidine ring mean plane is perpendicular to the dichlorophenyl ring with a dihedral angle of 81.66 (9)°. The carbonyl group, C10═O2, and the benzyl ring (C18-C24) ring have an (+)anti-clinal conformation with torsion angle (C18—C17—C25—O2) of 146.81 (16)°.

In the benzene ring (C11—C16) of the indole ring system, the expansion of the ipso angles at C11, C13 and C14 [121.71 (19), 121.1 (2) and 120.8 (2)°, respectively] and contraction of the apical angles at C12, C15 and C16 [117.9 (2), 119.13 (18) and 119.43 (16)°, respectively] are caused by the fusion of the smaller pyrrole ring to the six-membered benzene ring and the strain is taken up by the angular distortion rather than by bond-length distortions (Allen, 1981). The carboxyl group and oxindole ring system are (-)anti-clinal to each other with torsion angle (C9—C17—C25—O2) of -92.85 (18)°.

In the crystal, molecules are linked via N-H···O hydrogen bonds forming *C*(7) chains running parallel to [1 0 -1]; see Fig. 2 and Table 1.

### 2. Experimental

A mixture of (*E*)-methyl 2-benzyl-3-(2,4-dichlorophenyl)acrylate (2 mmol), isatin (2 mmol) and sarcosine (2 mmol) in acetonitrile (8 ml) was refluxed for 12 h. After the completion of the reaction as indicated by TLC, the reaction mixture was concentrated. The resulting crude mass was diluted with water (10 ml) and extracted with ethyl acetate (3 × 10 ml). The combined organic layers were washed with brine (2 × 10 ml) and dried over anhydrous Na_2_SO_4_. The organic layer was concentrated and the residue purified by column chromatography on silica gel (Acme 100–200 mesh), using ethyl acetate:hexanes (2:8) to afford the title compound as a colourless solid in (65%) yield. Block-like colourless crystals were obtained by slow evaporation of a solution in CHCl_3_.

### 3. Refinement

The H atoms could all be located in difference electron-density maps. In the final cycles of refinement they were treated as riding atoms and their distances were geometrically constrained: N-H = 0.86 Å, C—H = 0.93 - 0.98 Å with *U*_iso_(H) = 1.5 *U*_eq_(C– methyl) and = 1.2*U*_eq_(N/C) for other H atoms.

### Crystal data

**Table:**

C_27_H_24_Cl_2_N_2_O_3_	*F*(000) = 1032
*M**_r_* = 495.38	*D*_x_ = 1.381 Mg m^−^^3^
Monoclinic, *P*2_1_/*n*	Mo *K*α radiation, λ = 0.71073 Å
Hall symbol: -p 2yn	Cell parameters from 6466 reflections
*a* = 12.7051 (5) Å	θ = 2.1–29.3°
*b* = 14.1724 (6) Å	µ = 0.31 mm^−^^1^
*c* = 14.0322 (6) Å	*T* = 293 K
β = 109.424 (2)°	Block, colorless
*V* = 2382.85 (17) Å^3^	0.30 × 0.28 × 0.25 mm
*Z* = 4	

### Data collection

**Table:**

Bruker Kappa APEXII CCD diffractometer	4356 reflections with *I* > 2σ(*I*)
Radiation source: fine-focus sealed tube	*R*_int_ = 0.028
Graphite monochromator	θ_max_ = 29.3°, θ_min_ = 2.1°
ω scans	*h* = −16→17
27865 measured reflections	*k* = −19→13
6466 independent reflections	*l* = −18→19

### Refinement

**Table:**

Refinement on *F*^2^	Primary atom site location: structure-invariant direct methods
Least-squares matrix: full	Secondary atom site location: difference Fourier map
*R*[*F*^2^ > 2σ(*F*^2^)] = 0.044	Hydrogen site location: inferred from neighbouring sites
*wR*(*F*^2^) = 0.124	H-atom parameters constrained
*S* = 1.00	*w* = 1/[σ^2^(*F*_o_^2^) + (0.0537*P*)^2^ + 0.8129*P*] where *P* = (*F*_o_^2^ + 2*F*_c_^2^)/3
6466 reflections	(Δ/σ)_max_ < 0.001
309 parameters	Δρ_max_ = 0.41 e Å^−^^3^
0 restraints	Δρ_min_ = −0.49 e Å^−^^3^

### Special details

**Table:**

Geometry. All e.s.d.'s (except the e.s.d. in the dihedral angle between two l.s. planes) are estimated using the full covariance matrix. The cell e.s.d.'s are taken into account individually in the estimation of e.s.d.'s in distances, angles and torsion angles; correlations between e.s.d.'s in cell parameters are only used when they are defined by crystal symmetry. An approximate (isotropic) treatment of cell e.s.d.'s is used for estimating e.s.d.'s involving l.s. planes.
Refinement. Refinement of *F*^2^ against ALL reflections. The weighted *R*-factor *wR* and goodness of fit *S* are based on *F*^2^, conventional *R*-factors *R* are based on *F*, with *F* set to zero for negative *F*^2^. The threshold expression of *F*^2^ > σ(*F*^2^) is used only for calculating *R*-factors(gt) *etc*. and is not relevant to the choice of reflections for refinement. *R*-factors based on *F*^2^ are statistically about twice as large as those based on *F*, and *R*- factors based on ALL data will be even larger.

### Fractional atomic coordinates and isotropic or equivalent isotropic displacement parameters (Å^2^)

**Table:**

	*x*	*y*	*z*	*U*_iso_*/*U*_eq_	
C1	0.58285 (14)	0.31080 (12)	0.42423 (13)	0.0422 (4)	
C2	0.66005 (15)	0.33021 (13)	0.37693 (14)	0.0483 (4)	
H2	0.7301	0.3019	0.3985	0.058*	
C3	0.63062 (15)	0.39240 (13)	0.29724 (15)	0.0484 (4)	
C4	0.52940 (16)	0.43704 (13)	0.26718 (15)	0.0507 (5)	
H4	0.5118	0.4809	0.2149	0.061*	
C5	0.45350 (15)	0.41603 (13)	0.31561 (14)	0.0459 (4)	
H5	0.3848	0.4465	0.2951	0.055*	
C6	0.47685 (13)	0.35069 (12)	0.39402 (12)	0.0393 (4)	
C7	0.39119 (14)	0.32433 (12)	0.44254 (12)	0.0389 (4)	
H7	0.4320	0.2961	0.5082	0.047*	
C8	0.32681 (16)	0.40830 (13)	0.46311 (15)	0.0502 (4)	
H8A	0.3672	0.4374	0.5275	0.060*	
H8B	0.3137	0.4553	0.4102	0.060*	
C9	0.18556 (13)	0.30092 (11)	0.38043 (11)	0.0346 (3)	
C10	0.12965 (15)	0.34886 (13)	0.27525 (12)	0.0426 (4)	
C11	0.00156 (14)	0.24664 (13)	0.29794 (14)	0.0449 (4)	
C12	−0.09784 (16)	0.19937 (16)	0.28444 (19)	0.0649 (6)	
H12	−0.1569	0.2036	0.2239	0.078*	
C13	−0.10630 (18)	0.14611 (16)	0.3634 (2)	0.0670 (6)	
H13	−0.1722	0.1139	0.3562	0.080*	
C14	−0.01904 (18)	0.13971 (14)	0.45262 (18)	0.0581 (5)	
H14	−0.0266	0.1033	0.5051	0.070*	
C15	0.08044 (15)	0.18687 (13)	0.46545 (14)	0.0446 (4)	
H15	0.1391	0.1829	0.5263	0.054*	
C16	0.09119 (13)	0.23953 (12)	0.38709 (12)	0.0373 (4)	
C17	0.29931 (12)	0.25062 (11)	0.38356 (11)	0.0311 (3)	
C18	0.29733 (13)	0.22452 (11)	0.27577 (11)	0.0339 (3)	
H18A	0.2346	0.1824	0.2471	0.041*	
H18B	0.2812	0.2819	0.2358	0.041*	
C19	0.39826 (12)	0.17915 (11)	0.25989 (11)	0.0334 (3)	
C20	0.42743 (15)	0.21109 (13)	0.17845 (13)	0.0447 (4)	
H20	0.3863	0.2595	0.1384	0.054*	
C21	0.51556 (17)	0.17291 (15)	0.15552 (16)	0.0561 (5)	
H21	0.5334	0.1958	0.1007	0.067*	
C22	0.57727 (16)	0.10121 (14)	0.21310 (15)	0.0522 (5)	
H22	0.6376	0.0759	0.1983	0.063*	
C23	0.54893 (15)	0.06731 (13)	0.29259 (14)	0.0464 (4)	
H23	0.5897	0.0180	0.3313	0.056*	
C24	0.46040 (14)	0.10554 (12)	0.31595 (12)	0.0401 (4)	
H24	0.4423	0.0814	0.3702	0.048*	
C25	0.30887 (12)	0.16134 (11)	0.44713 (11)	0.0331 (3)	
C26	0.25651 (17)	0.00191 (13)	0.44542 (15)	0.0532 (5)	
H26A	0.3325	−0.0180	0.4765	0.080*	
H26B	0.2161	−0.0455	0.3986	0.080*	
H26C	0.2226	0.0109	0.4965	0.080*	
C27	0.13946 (19)	0.43922 (15)	0.46547 (17)	0.0609 (5)	
H27A	0.1663	0.4751	0.5269	0.091*	
H27B	0.0706	0.4089	0.4616	0.091*	
H27C	0.1273	0.4806	0.4086	0.091*	
N1	0.22212 (12)	0.36773 (10)	0.46454 (10)	0.0426 (3)	
N2	0.02779 (13)	0.31006 (12)	0.23303 (11)	0.0521 (4)	
H2A	−0.0160	0.3229	0.1730	0.063*	
O1	0.16988 (12)	0.41046 (10)	0.23798 (10)	0.0562 (4)	
O2	0.35713 (10)	0.15537 (9)	0.53672 (8)	0.0471 (3)	
O3	0.25399 (9)	0.08941 (8)	0.39242 (8)	0.0379 (3)	
Cl1	0.62311 (4)	0.23046 (4)	0.52348 (4)	0.06078 (16)	
Cl2	0.72403 (5)	0.41173 (4)	0.23263 (5)	0.07259 (19)	

### Atomic displacement parameters (Å^2^)

**Table:**

	*U*^11^	*U*^22^	*U*^33^	*U*^12^	*U*^13^	*U*^23^
C1	0.0388 (9)	0.0436 (9)	0.0367 (9)	−0.0069 (7)	0.0025 (7)	0.0008 (7)
C2	0.0367 (9)	0.0496 (11)	0.0534 (11)	−0.0066 (7)	0.0082 (8)	−0.0034 (8)
C3	0.0468 (10)	0.0445 (10)	0.0570 (11)	−0.0149 (8)	0.0214 (9)	−0.0047 (8)
C4	0.0542 (11)	0.0432 (10)	0.0550 (11)	−0.0088 (8)	0.0185 (9)	0.0090 (8)
C5	0.0411 (9)	0.0446 (10)	0.0488 (10)	−0.0032 (7)	0.0106 (8)	0.0072 (8)
C6	0.0371 (8)	0.0400 (9)	0.0355 (8)	−0.0079 (7)	0.0052 (7)	0.0000 (7)
C7	0.0380 (8)	0.0435 (9)	0.0300 (8)	−0.0073 (7)	0.0044 (6)	0.0007 (7)
C8	0.0594 (11)	0.0451 (10)	0.0478 (11)	−0.0102 (8)	0.0201 (9)	−0.0114 (8)
C9	0.0356 (8)	0.0392 (8)	0.0281 (8)	0.0044 (6)	0.0094 (6)	0.0030 (6)
C10	0.0469 (10)	0.0471 (10)	0.0342 (9)	0.0186 (8)	0.0141 (7)	0.0067 (7)
C11	0.0382 (9)	0.0475 (10)	0.0444 (10)	0.0092 (7)	0.0076 (8)	−0.0071 (8)
C12	0.0387 (10)	0.0653 (14)	0.0781 (16)	0.0025 (9)	0.0025 (10)	−0.0212 (12)
C13	0.0462 (11)	0.0567 (13)	0.1007 (19)	−0.0084 (9)	0.0278 (12)	−0.0159 (13)
C14	0.0586 (12)	0.0504 (11)	0.0786 (15)	−0.0027 (9)	0.0406 (12)	−0.0035 (10)
C15	0.0450 (9)	0.0482 (10)	0.0463 (10)	0.0033 (8)	0.0229 (8)	−0.0004 (8)
C16	0.0338 (8)	0.0415 (9)	0.0371 (9)	0.0057 (6)	0.0125 (7)	−0.0040 (7)
C17	0.0290 (7)	0.0365 (8)	0.0245 (7)	0.0008 (6)	0.0043 (6)	0.0029 (6)
C18	0.0326 (8)	0.0417 (9)	0.0248 (7)	0.0037 (6)	0.0060 (6)	0.0039 (6)
C19	0.0315 (7)	0.0379 (8)	0.0286 (7)	−0.0015 (6)	0.0071 (6)	−0.0019 (6)
C20	0.0495 (10)	0.0466 (10)	0.0420 (10)	0.0084 (8)	0.0205 (8)	0.0100 (8)
C21	0.0635 (12)	0.0614 (12)	0.0562 (12)	0.0072 (10)	0.0369 (10)	0.0125 (10)
C22	0.0440 (10)	0.0588 (12)	0.0603 (12)	0.0069 (8)	0.0263 (9)	−0.0008 (9)
C23	0.0416 (9)	0.0478 (10)	0.0469 (10)	0.0102 (8)	0.0110 (8)	0.0038 (8)
C24	0.0414 (9)	0.0454 (10)	0.0338 (8)	0.0041 (7)	0.0128 (7)	0.0053 (7)
C25	0.0282 (7)	0.0409 (8)	0.0272 (7)	0.0022 (6)	0.0053 (6)	0.0019 (6)
C26	0.0685 (13)	0.0385 (10)	0.0508 (11)	−0.0036 (9)	0.0175 (10)	0.0076 (8)
C27	0.0754 (14)	0.0522 (12)	0.0647 (13)	0.0093 (10)	0.0362 (11)	−0.0085 (10)
N1	0.0498 (8)	0.0424 (8)	0.0365 (8)	0.0002 (6)	0.0156 (6)	−0.0062 (6)
N2	0.0453 (9)	0.0654 (10)	0.0348 (8)	0.0157 (7)	−0.0012 (6)	0.0029 (7)
O1	0.0649 (9)	0.0567 (8)	0.0516 (8)	0.0211 (7)	0.0253 (7)	0.0221 (6)
O2	0.0499 (7)	0.0546 (7)	0.0272 (6)	−0.0063 (6)	0.0000 (5)	0.0084 (5)
O3	0.0422 (6)	0.0362 (6)	0.0320 (6)	−0.0011 (5)	0.0079 (5)	0.0016 (4)
Cl1	0.0510 (3)	0.0749 (4)	0.0453 (3)	0.0069 (2)	0.0010 (2)	0.0180 (2)
Cl2	0.0699 (4)	0.0693 (4)	0.0948 (5)	−0.0121 (3)	0.0490 (3)	0.0052 (3)

### Geometric parameters (Å, º)

**Table:**

C1—C2	1.383 (3)	C14—C15	1.388 (3)
C1—C6	1.390 (2)	C14—H14	0.9300
C1—Cl1	1.7389 (18)	C15—C16	1.373 (2)
C2—C3	1.374 (3)	C15—H15	0.9300
C2—H2	0.9300	C17—C25	1.530 (2)
C3—C4	1.368 (3)	C17—C18	1.549 (2)
C3—Cl2	1.7381 (19)	C18—C19	1.516 (2)
C4—C5	1.384 (2)	C18—H18A	0.9700
C4—H4	0.9300	C18—H18B	0.9700
C5—C6	1.393 (2)	C19—C24	1.385 (2)
C5—H5	0.9300	C19—C20	1.389 (2)
C6—C7	1.510 (2)	C20—C21	1.375 (3)
C7—C8	1.525 (3)	C20—H20	0.9300
C7—C17	1.580 (2)	C21—C22	1.371 (3)
C7—H7	0.9800	C21—H21	0.9300
C8—N1	1.455 (2)	C22—C23	1.368 (3)
C8—H8A	0.9700	C22—H22	0.9300
C8—H8B	0.9700	C23—C24	1.382 (2)
C9—N1	1.463 (2)	C23—H23	0.9300
C9—C16	1.509 (2)	C24—H24	0.9300
C9—C10	1.564 (2)	C25—O2	1.2045 (18)
C9—C17	1.599 (2)	C25—O3	1.3262 (19)
C10—O1	1.214 (2)	C26—O3	1.441 (2)
C10—N2	1.348 (2)	C26—H26A	0.9600
C11—C12	1.386 (3)	C26—H26B	0.9600
C11—C16	1.387 (2)	C26—H26C	0.9600
C11—N2	1.397 (3)	C27—N1	1.462 (2)
C12—C13	1.374 (3)	C27—H27A	0.9600
C12—H12	0.9300	C27—H27B	0.9600
C13—C14	1.372 (3)	C27—H27C	0.9600
C13—H13	0.9300	N2—H2A	0.8600
			
C2—C1—C6	122.86 (16)	C15—C16—C9	130.97 (15)
C2—C1—Cl1	116.62 (14)	C11—C16—C9	109.45 (15)
C6—C1—Cl1	120.50 (13)	C25—C17—C18	110.14 (12)
C3—C2—C1	118.27 (17)	C25—C17—C7	109.87 (12)
C3—C2—H2	120.9	C18—C17—C7	116.02 (13)
C1—C2—H2	120.9	C25—C17—C9	106.29 (12)
C4—C3—C2	121.40 (17)	C18—C17—C9	111.02 (11)
C4—C3—Cl2	120.08 (15)	C7—C17—C9	102.90 (12)
C2—C3—Cl2	118.51 (15)	C19—C18—C17	120.33 (12)
C3—C4—C5	119.09 (17)	C19—C18—H18A	107.2
C3—C4—H4	120.5	C17—C18—H18A	107.2
C5—C4—H4	120.5	C19—C18—H18B	107.2
C4—C5—C6	122.06 (17)	C17—C18—H18B	107.2
C4—C5—H5	119.0	H18A—C18—H18B	106.9
C6—C5—H5	119.0	C24—C19—C20	117.08 (15)
C1—C6—C5	116.20 (16)	C24—C19—C18	125.91 (14)
C1—C6—C7	122.15 (15)	C20—C19—C18	116.92 (14)
C5—C6—C7	121.65 (15)	C21—C20—C19	121.67 (17)
C6—C7—C8	113.87 (14)	C21—C20—H20	119.2
C6—C7—C17	116.42 (13)	C19—C20—H20	119.2
C8—C7—C17	105.41 (13)	C22—C21—C20	120.30 (17)
C6—C7—H7	106.9	C22—C21—H21	119.8
C8—C7—H7	106.9	C20—C21—H21	119.8
C17—C7—H7	106.9	C23—C22—C21	119.14 (17)
N1—C8—C7	104.15 (14)	C23—C22—H22	120.4
N1—C8—H8A	110.9	C21—C22—H22	120.4
C7—C8—H8A	110.9	C22—C23—C24	120.74 (17)
N1—C8—H8B	110.9	C22—C23—H23	119.6
C7—C8—H8B	110.9	C24—C23—H23	119.6
H8A—C8—H8B	108.9	C23—C24—C19	121.04 (16)
N1—C9—C16	111.63 (12)	C23—C24—H24	119.5
N1—C9—C10	113.75 (13)	C19—C24—H24	119.5
C16—C9—C10	100.84 (13)	O2—C25—O3	122.50 (14)
N1—C9—C17	102.99 (12)	O2—C25—C17	125.52 (14)
C16—C9—C17	118.08 (13)	O3—C25—C17	111.95 (12)
C10—C9—C17	110.01 (12)	O3—C26—H26A	109.5
O1—C10—N2	125.73 (16)	O3—C26—H26B	109.5
O1—C10—C9	126.47 (17)	H26A—C26—H26B	109.5
N2—C10—C9	107.80 (15)	O3—C26—H26C	109.5
C12—C11—C16	121.71 (19)	H26A—C26—H26C	109.5
C12—C11—N2	128.77 (18)	H26B—C26—H26C	109.5
C16—C11—N2	109.43 (16)	N1—C27—H27A	109.5
C13—C12—C11	117.9 (2)	N1—C27—H27B	109.5
C13—C12—H12	121.1	H27A—C27—H27B	109.5
C11—C12—H12	121.1	N1—C27—H27C	109.5
C14—C13—C12	121.1 (2)	H27A—C27—H27C	109.5
C14—C13—H13	119.5	H27B—C27—H27C	109.5
C12—C13—H13	119.5	C8—N1—C27	112.88 (15)
C13—C14—C15	120.8 (2)	C8—N1—C9	106.92 (13)
C13—C14—H14	119.6	C27—N1—C9	114.79 (14)
C15—C14—H14	119.6	C10—N2—C11	112.22 (14)
C16—C15—C14	119.13 (18)	C10—N2—H2A	123.9
C16—C15—H15	120.4	C11—N2—H2A	123.9
C14—C15—H15	120.4	C25—O3—C26	116.46 (13)
C15—C16—C11	119.43 (16)		
			
C6—C1—C2—C3	0.7 (3)	C8—C7—C17—C18	118.92 (15)
Cl1—C1—C2—C3	179.12 (14)	C6—C7—C17—C9	−129.76 (14)
C1—C2—C3—C4	2.4 (3)	C8—C7—C17—C9	−2.49 (16)
C1—C2—C3—Cl2	−176.21 (14)	N1—C9—C17—C25	93.64 (13)
C2—C3—C4—C5	−2.7 (3)	C16—C9—C17—C25	−29.85 (17)
Cl2—C3—C4—C5	175.91 (15)	C10—C9—C17—C25	−144.76 (13)
C3—C4—C5—C6	−0.1 (3)	N1—C9—C17—C18	−146.58 (13)
C2—C1—C6—C5	−3.2 (3)	C16—C9—C17—C18	89.92 (16)
Cl1—C1—C6—C5	178.40 (13)	C10—C9—C17—C18	−24.99 (18)
C2—C1—C6—C7	176.31 (16)	N1—C9—C17—C7	−21.83 (14)
Cl1—C1—C6—C7	−2.1 (2)	C16—C9—C17—C7	−145.33 (13)
C4—C5—C6—C1	2.9 (3)	C10—C9—C17—C7	99.76 (14)
C4—C5—C6—C7	−176.61 (17)	C25—C17—C18—C19	−64.72 (18)
C1—C6—C7—C8	137.44 (17)	C7—C17—C18—C19	60.85 (19)
C5—C6—C7—C8	−43.1 (2)	C9—C17—C18—C19	177.83 (13)
C1—C6—C7—C17	−99.58 (18)	C17—C18—C19—C24	45.3 (2)
C5—C6—C7—C17	79.9 (2)	C17—C18—C19—C20	−138.30 (16)
C6—C7—C8—N1	155.06 (14)	C24—C19—C20—C21	−1.4 (3)
C17—C7—C8—N1	26.25 (17)	C18—C19—C20—C21	−178.13 (17)
N1—C9—C10—O1	54.9 (2)	C19—C20—C21—C22	0.2 (3)
C16—C9—C10—O1	174.51 (16)	C20—C21—C22—C23	1.0 (3)
C17—C9—C10—O1	−60.1 (2)	C21—C22—C23—C24	−1.0 (3)
N1—C9—C10—N2	−124.69 (15)	C22—C23—C24—C19	−0.2 (3)
C16—C9—C10—N2	−5.07 (16)	C20—C19—C24—C23	1.3 (2)
C17—C9—C10—N2	120.37 (14)	C18—C19—C24—C23	177.76 (16)
C16—C11—C12—C13	1.3 (3)	C18—C17—C25—O2	146.81 (16)
N2—C11—C12—C13	−174.74 (19)	C7—C17—C25—O2	17.8 (2)
C11—C12—C13—C14	−0.2 (3)	C9—C17—C25—O2	−92.85 (18)
C12—C13—C14—C15	−0.1 (3)	C18—C17—C25—O3	−35.14 (17)
C13—C14—C15—C16	−0.7 (3)	C7—C17—C25—O3	−164.13 (12)
C14—C15—C16—C11	1.8 (2)	C9—C17—C25—O3	85.20 (14)
C14—C15—C16—C9	176.90 (16)	C7—C8—N1—C27	−169.79 (15)
C12—C11—C16—C15	−2.2 (3)	C7—C8—N1—C9	−42.64 (17)
N2—C11—C16—C15	174.60 (15)	C16—C9—N1—C8	167.99 (14)
C12—C11—C16—C9	−178.24 (16)	C10—C9—N1—C8	−78.71 (17)
N2—C11—C16—C9	−1.49 (19)	C17—C9—N1—C8	40.32 (16)
N1—C9—C16—C15	−50.5 (2)	C16—C9—N1—C27	−65.99 (18)
C10—C9—C16—C15	−171.62 (17)	C10—C9—N1—C27	47.31 (19)
C17—C9—C16—C15	68.6 (2)	C17—C9—N1—C27	166.33 (14)
N1—C9—C16—C11	125.01 (14)	O1—C10—N2—C11	−174.94 (16)
C10—C9—C16—C11	3.87 (16)	C9—C10—N2—C11	4.64 (19)
C17—C9—C16—C11	−115.93 (15)	C12—C11—N2—C10	174.33 (18)
C6—C7—C17—C25	117.36 (15)	C16—C11—N2—C10	−2.1 (2)
C8—C7—C17—C25	−115.37 (14)	O2—C25—O3—C26	−1.2 (2)
C6—C7—C17—C18	−8.4 (2)	C17—C25—O3—C26	−179.28 (14)

### Hydrogen-bond geometry (Å, º)

**Table:**

*D*—H···*A*	*D*—H	H···*A*	*D*···*A*	*D*—H···*A*
C18—H18*B*···O1	0.97	2.31	3.046 (2)	132
C24—H24···O3	0.93	2.52	3.155 (2)	126
N2—H2*A*···O2^i^	0.86	2.07	2.924 (2)	170

Symmetry code: (i) *x*−1/2, −*y*+1/2, *z*−1/2.
